# Early origin and evolution of the FtsZ/tubulin protein family

**DOI:** 10.3389/fmicb.2022.1100249

**Published:** 2023-01-10

**Authors:** Carlos Santana-Molina, DMaría del Saz-Navarro, Damien P. Devos

**Affiliations:** ^1^Centro Andaluz de Biología del Desarrollo, Consejo Superior de Investigaciones Científicas, Junta de Andalucía, Universidad Pablo de Olavide, Seville, Spain; ^2^Department of Marine Microbiology and Biogeochemistry, Royal Netherlands Institute for Sea Research (NIOZ), University of Utrecht, Utrecht, Netherlands

**Keywords:** FtsZ, tubulin, CetZ, evolution, tree of life

## Abstract

The origin of the FtsZ/tubulin protein family was extremely relevant for life since these proteins are present in nearly all organisms, carrying out essential functions such as cell division or forming a major part of the cytoskeleton in eukaryotes. Therefore, investigating the early evolution of the FtsZ/tubulin protein family could reveal crucial aspects of the diversification of the three domains of life. In this study, we revisited the phylogenies of the FtsZ/tubulin protein family in an extensive prokaryotic diversity, focusing on the main evolutionary events that occurred during its evolution. We found evidence of its early origin in the last universal common ancestor since FtsZ was present in the last common ancestor of Bacteria and Archaea. In bacteria, *ftsZ* genes are genomically associated with the bacterial division gene cluster, while in archaea, *ftsZ* duplicated prior to archaeal diversification, and one of the copies is associated with protein biosynthesis genes. Archaea have expanded the FtsZ/tubulin protein family with sequences closely related to eukaryotic tubulins. In addition, we report novel CetZ-like groups in Halobacterota and Asgardarchaeota. Investigating the C-termini of prokaryotic paralogs basal to eukaryotic tubulins, we show that archaeal CetZ, as well as the plasmidic TubZ from Firmicutes, most likely originated from archaeal FtsZ. Finally, prokaryotic tubulins are restricted to Odinarchaeaota and *Prosthecobacter* species, and they seem to belong to different molecular systems. However, their phylogenies suggest that they are closely related to α/β-tubulins pointing to a potential ancestrality of these eukaryotic paralogs of tubulins.

## Introduction

Members of the FtsZ/tubulin protein family are small GTPases that orchestrate essential cellular processes such as cell division in prokaryotes and eukaryotes or establishing the cytoskeleton in eukaryotes (Wagstaff and Löwe, 2018). These proteins are present in most organisms in the three domains of life, with few exceptions. Therefore, their omnipresence and fundamental functions in living organisms indicate the relevance of the FtsZ/tubulin protein family for the origin and diversification of life.

In prokaryotes, FtsZ polymerizes to form a ring in the septal region of dividing cells, recruiting and cooperating with other proteins to carry out the process of division (Bi and Lutkenhaus, 1991). In Bacteria, FtsZ cooperates with the so-called bacterial divisome, which comprises proteins also found in Archaea such as FtsA/Mreb or SepF, and other proteins exclusively found in bacteria, which are mostly involved in peptidoglycan metabolism (whose genes form the *dcw* gene cluster; Ayala et al., 1994). Archaea usually present two ancestral copies of FtsZ that appear to have non-overlapping functions (Liao et al., 2021). The cell division process in Archaea is not as well understood as in Bacteria, but there are some similarities with the bacterial machinery, for example, FtsA and SepF (Pende et al., 2021; van Wolferen et al., 2022). Alternatively, some archaea that do not have FtsZ appear to have a cell division system based on ESCRT-III homologs (Caspi and Dekker, 2018; Pulschen et al., 2020). However, there are specific cases in prokaryotes that do not have FtsZ (nor ESCRT-III), and whose cell division mechanisms remain unknown (Lluch-Senar et al., 2010; Rivas-Marín et al., 2016; Ithurbide et al., 2022). On the other side, the tubulins constitute one of the main components of the eukaryotic cytoskeleton, which has been expanded in different paralogs with specific functions (Janke and Magiera, 2020). α- and β-tubulins heterodimerize, forming the canonical microtubules, while γ-tubulin is involved in their nucleation. δ-, ε-, and ζ-tubulins constitute the centriole controlling chromosome segregation during cell division. Thus, the members of the FtsZ/tubulin protein family have diversified functionally across the three domains of life, but they retain essential functions in cell division.

**FIGURE 1:**
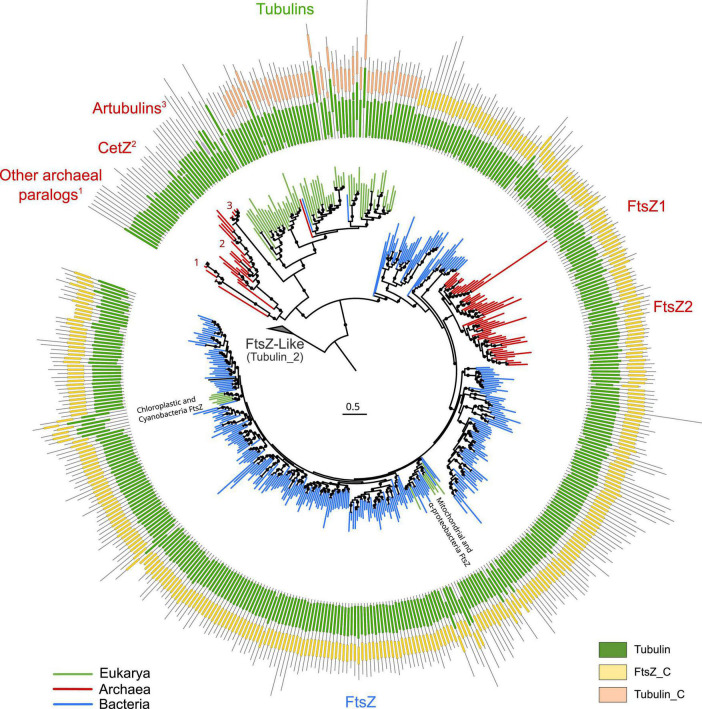
Overview of the FtsZ/tubulin protein family. Schematic phylogeny of representative sequences showing their Pfam domain architectures. Branches are colored according to the taxonomic domain, and black circles indicate bootstraps higher than 90. The tree was rooted at the divergent FtsZ-like sequences containing the Tubulin_2 Pfam domain. Note that some sequences discussed in this study are not shown in this tree (e.g., TubZ and other archaeal paralogs).

To provide a global overview of the evolution of the FtsZ/tubulin protein family, we reconstructed a phylogeny of representative sequences representing the diversity of subfamilies, their relationships, and their domain architecture (Figure 1). The FtsZ/tubulin protein family is mainly composed of prokaryotic FtsZ [including mitochondrial (Leger et al., 2015) and chloroplastic (TerBush et al., 2013) ones], eukaryotic tubulins [including prokaryotic sequences from Asgardarchaeota (Akıl et al., 2022) and Verrucomicrobia (Schlieper et al., 2005)], archaeal paralogs closely related to tubulins [including CetZ (Duggin et al., 2015), artubulins (Yutin and Koonin, 2012), and other bacterial and archaeal sequences not included in this tree such as the plasmidic TubZ found in Firmicutes (Larsen et al., 2007)], and divergent homologs bearing the tubulin_2 Pfam domain, which were used to root the tree. This latter group, here called FtsZ-like, displayed diverse domain composition and poorly aligned positions, and given its sparse distribution in prokaryotes, domain architecture, and genomic synteny, its evolutionary history is unlikely to be directly related to the one of the FtsZ/tubulin protein family (Makarova and Koonin, 2010). Similarly, the divergent eukaryotic proteins related to tubulins called Misato (not included in this tree) appear to be a eukaryotic subfunctionalization given that they show various motif extensions (Mottier-Pavie et al., 2011; Palumbo et al., 2015).

The domain architecture of these proteins shows a conserved N-terminus, while the C-termini have diverged between the subfamilies: FtsZ sequences have the FtsZ_C domain, and tubulins have tubulin_C domain (not in all paralogs). Remarkably, the archaeal paralogs basal to tubulins, including CetZ, did not present any recognizable Pfam domain. These observations on the C-terminus domains suggest a dynamic evolutionary region whose evolution could clarify the relationships between the members of these protein families. This is important because the evolutionary relationships between some of these prokaryotic proteins and eukaryotic tubulins are still debated.

Given the relevance of the FtsZ/tubulin protein family, a comprehensive understanding of its evolution would provide crucial insights into the diversification of the three domains of life. FtsZ is expected to have originated in the last universal common ancestor (LUCA) since it is in nearly all Bacteria and Archaea (Davis, 2002), both forming the two primary domains of life. On the other hand, tubulins are expected to have originated from multiple duplications during eukaryogenesis between the first eukaryotic common ancestor (FECA) and the last eukaryotic common ancestor (LECA), resulting in the contemporary α, β, δ-, ε-, and ζ-tubulins [possibly among others; (Findeisen et al., 2014)]. However, to the best of our knowledge, questions about the origin of tubulins and the order of their duplications are still limited. The identification of artubulins found in the *Nitrosoarcheaum* genus led to the suggestion of the archaeal origin of tubulins (Yutin and Koonin, 2012). However, posterior analyses suggested alternative scenarios given the phylogenetic relationships of artubulins with TubZ (Findeisen et al., 2014). Therefore, there are some controversies around the origin of some of these proteins that need to be addressed. In addition, greater taxonomic diversity and improved knowledge about the tree of life (Coleman et al., 2021; Moody et al., 2022) will help to decipher the main events during the evolution of the FtsZ/tubulin protein family.

In this study, we revisit the evolutionary history of the FtsZ/tubulin protein family, focusing on the main events in its evolution: (i) the early evolution of FtsZ in Bacteria and Archaea, (ii) the origins of archaeal paralogs found basal to tubulins based on their C-termini, and (iii) the relationships between eukaryotic tubulins and prokaryotic tubulins. Addressing these conundrums, we provide evidence for the early origin and evolution of this protein family and clarify some relationships of archaeal paralogs, showing that the C-termini of CetZ and TubZ relate their origin with archaeal FtsZ.

## Results

### FtsZ was present in the last bacterial common ancestor and was genomically associated with the bacterial divisome

Two datasets were built, one including bacterial and archaeal FtsZs (BA reconstruction) and the other including only the bacterial sequences (B reconstruction). After removing redundancy by taxonomic classes and spurious sequences, the final multiple sequence alignments (MSAs) were treated with three different trimming methods, referred to as Manual-, BMGE-, and trimAl-gt70-trimming (refer to section “Materials and methods”). Subsequently, we performed phylogenetic reconstructions using empirical models automatically selected by Modelfinder (Kalyaanamoorthy et al., 2017) and complex models (LG4X and C20 + R + F). We used an ultrafast bootstrap for manual-trimmed reconstructions and nearest neighbor interchange (NNI) search analyses (Minh et al., 2020) for all the MSAs.

**FIGURE 2:**
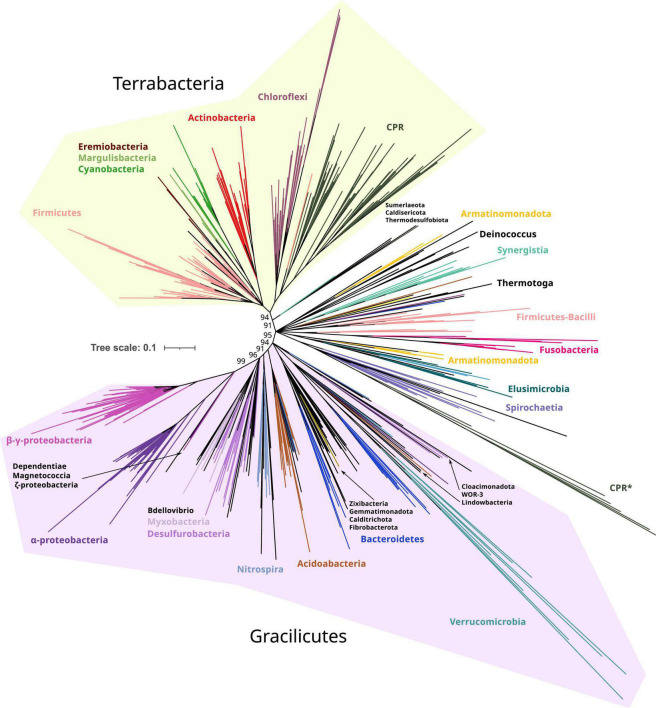
Phylogeny of only bacterial FtsZs. Branches are colored according to taxonomic phyla and GTDB nomenclature (release bac120, arc122). The dataset was reduced by redundant filtering by taxonomic classes and then selecting one sequence for each taxonomic order. The final MSA consisted of 534 sequences and 334 positions, denoted as manual trimming. The phylogeny was built using IQ-TREE, automatic model selection (LG + R10), and ultrafast bootstrap (UFBoot2). Refer to Supplementary Figure 1, for corrected bootstrap trees with NNI searches, different trimming methods, and evolutionary models. To ease the visualization of the most basal supported nodes, nodes with lower support than 85% were removed and only UFBoot2 values at basal nodes of interest were shown. Terrabacteria and Gracilicutes supergroups are highlighted in yellow and purple, respectively (refer to the extended tree in Supplementary Figure 3). Original and trimmed analyses and Newick trees are provided in Supplementary Data.

In the resulting reconstructions, most bacterial phyla show a monophyletic pattern, although the topology was sensitive to different parameters, such as taxonomic sampling, sequence redundancy threshold, and phylogenetic methods (Supplementary Figure 1). Basal nodes were weakly supported (<90%) in most reconstructions, except for UFBoot2 reconstructions. This low support in basal nodes can be explained by the antiquity and short length of these proteins (MSAs of 350 amino acids applying inclusive trimming). Therefore, despite the fact that UFBoot2 values lower than 95% should not be considered reliable nodes (Hoang et al., 2018), we interpreted these reconstructions with a relaxed threshold of confidence as the biological relationship between species is limited when collapsing nodes with 95% UFBoot2 values (Supplementary Figure 2). On the contrary, collapsing at 85% resulted in biologically meaningful clades (Figure 2 and refer to Supplementary Figures 2, 3 for an extended version).

Taking the Manual + UFBoot2 + MF reconstruction as a reference, the phylogeny was better resolved in the B reconstruction than in the BA reconstruction since the basal nodes better resolved the relationships between bacterial phyla (specifically in Gracilicutes, Supplementary Figure 2). This suggests that the addition of archaeal sequences promotes long branch attraction (LBA) artifacts, providing weaker topologies. This was observed in our reconstructions where archaeal FtsZ sequences attracted different bacterial FtsZ using various trimming methods and evolutionary models (refer to BA reconstructions in Supplementary Figure 1). Thus, B reconstruction was selected as a reference (Figure 2).

The bacterial FtsZ tree recovered the two main bacterial supergroups, Terrabacteria and Gracilicutes (Figure 2; Coleman et al., 2021). Between these two supergroups, intermediary branches are found, including Elusimicrobiota, Spirochaetota, Fusobacteriota, Synergistota, Armatimonadota, and Deinococcota phyla, or other phyla such as Firmicutes and Bacteroidota, forming paraphyletic groups and showing unstable locations in our reconstructions (Supplementary Figure 1). The FtsZ of Candidate Phyla Radiation (CPR, denoted as Patescibacteria in this GTDB version) is phylogenetically associated with Chloroflexota, consistent with their proposed origin (Coleman et al., 2021); however, this relationship was only observed in Manual-trimming reconstructions. Some CPR genomes also contain a divergent copy of FtsZ, which usually branches with Verrucomicrobiota, which are the most divergent groups (Figure 2 and Supplementary Figure 1). The verrucomicrobial FtsZ and the second copy of CPR had different topologies in the BA and B reconstructions (Supplementary Figures 1, 2), demonstrating their phylogenetic instability and suggesting strong divergence and artifacts usually associated with it, such as LBA. The divergent CPR FtsZ (together with that of Verrucomicrobiota) is nested in the canonical CPR FtsZ group in the BMGE-B-bnni reconstruction (Supplementary Figure 1), suggesting that it originated from the original FtsZ of CPR. In contrast, in the B reconstruction (refer to Figure 2 for UFBoot2 and Supplementary Figure 1 for NNI analysis), Verrucomicrobiota branches with other Gracilicutes near to Bacteroidetes (and other close relatives such as Cloacimonadota and WOR-3), consistent with their species relationship and supporting the idea that reconstructions without archaeal sequences solve more adequately bacterial evolution. Therefore, despite the phylogenetic instabilities or strong sequence divergence, the separation of Gracilicutes and Terrabacteria in most reconstructions (Supplementary Figures 1, 2 and in Manual-B-UFboot2 reconstruction supported by 95%; Figure 2) strongly suggests that FtsZ was present in the last bacterial common ancestor (LBCA).

**FIGURE 3:**
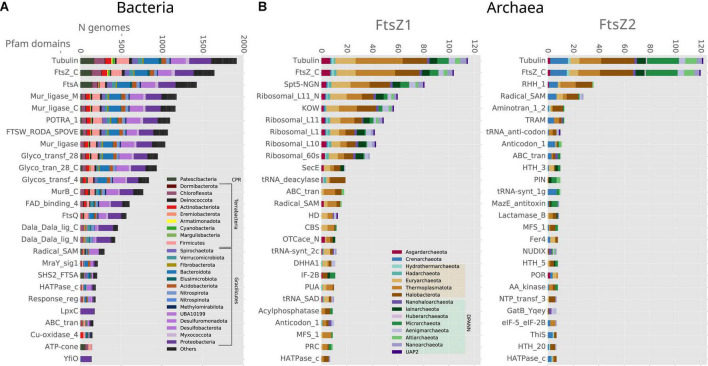
Genomic context analyses of the prokaryotic *ftsZ* genes. Analyses of **(A)** bacterial and **(B)** archaeal *ftsZ* genes, showing the 25 Pfam domains most abundant in their genome context; 10 Kb up and downstream of the *ftsZ* gene. Bars denote the number of genomes of each phylum, having at least one Pfam domain in the respective genome context. Note that some Pfam domains belong to the same proteins, such as Tubulin-FtsZ_C, Potra_1-FtsQ, and Mur_ligase_M/C.

We then inspected the genomic association of all these prokaryotic FtsZs (Figure 3). The most conserved Pfam domains present in the genes surrounding bacterial *ftsZs* are related to cell division, for example, FtsA, FtsW, or FtsQ, or peptidoglycan synthesis, for example, murein ligase, glycosyl transport, MreB, or MraY (Figure 3A), which form the *dcw* cluster. This genome context is well conserved in Gracilicutes, Terrabacteria, and CPR, showing that the *dcw* gene cluster, was already established in the LBCA. In the few bacteria having two copies of the *ftsZ* gene, such as Bipolaricuta, the additional copy is located outside the *dcw* cluster (some of them in conserved *loci*), indicating possible subfunctionalization due to gene duplication.

### FtsZ duplicated in the last archaeal common ancestor and was genomically associated with protein biosynthesis genes

**FIGURE 4:**
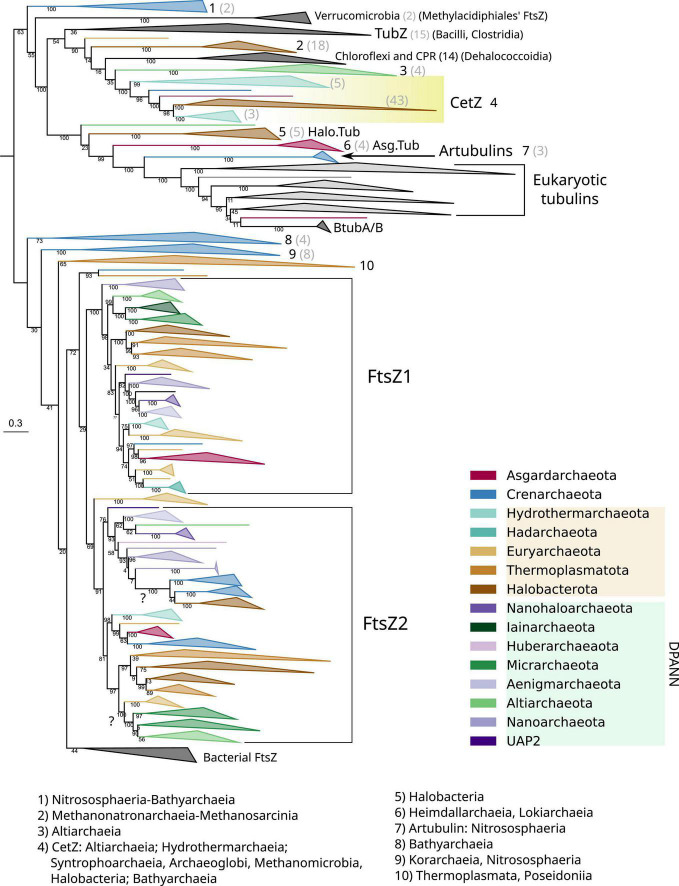
Phylogeny of all members of the FtsZ/tubulin protein family centered on archaeal sequences. The tree was rooted at the mid-point. Branches are colored according to taxonomic phyla following GTDB nomenclature (release bac120, arc122). The dataset was constructed by a selection of representative bacterial FtsZs and eukaryotic tubulins, plus an archaeal selection based on redundancy filtering and adding back removed paralogs from the remaining taxa. The final MSA consisted of 1,637 sequences and 410 positions, trimmed with trimAl *-*gt 0.2. The phylogeny was built using IQ-TREE, automatic model selection (LG + R10), and ultrafast bootstrap (UFBoot2). Numbers indicate the taxonomic composition of each phylogenetic group shown at the bottom, and gray numbers between parenthesis are the number of sequences for the respective group. Refer to the extended tree in Supplementary Figure 4.

The genomes of most archaeal species present two copies of *ftsZ*, coding for FtsZ1 and FtsZ2, with the exception of Crenarchaeota archaea that mostly retained FtsZ2, or present divergent FtsZs branching outside of the canonical archaeal FtsZ groups (Figure 4 and Supplementary Figure 4, for an extended view). Alternatively, other Crenarchaeota classes do not present any FtsZ, and in the case of a few Euryarchaeota and a few Asgardarchaeota, only one copy of the gene was found, as previously reported (Pende et al., 2021).

The Crenarchaeota and Asgardarchaeota sequences are closely related (Figure 4), in agreement with their species relationships. The Euryarchaeota sequences (according to GTDB) tend to form monophyletic groups, but they are not monophyletic with other close relatives such as Halobacterota and Thermoplasmatota. However, Hydrothermarchaeota and Hadarchaeota sequences are closely related to Euryarchaeota (Figure 4), which is in agreement with the species’ relationships. The DPANN supergroup branches paraphyletically: Micrarchaeota and Altiarchaeota branch close to the Euryarchaeota supergroup but distally to other DPANN such as Nanoarchaeota and Huberarchaeaota that show different topologies of the FtsZ1 and FtsZ2 branches.

Acknowledging the low resolution in some basal nodes, and as previously suggested (Pende et al., 2021), the monophyletic branching of most archaeal phyla, combined with topological similarities with the archaeal species tree, suggests that FtsZ1 and FtsZ2 are derived from an ancestral duplication in the last archaeal common ancestor (LACA). In addition, FtsZ evolution in archaea is irregular as some archaeal groups have lost one or both FtsZ copies, for example, Crenarchaeota. However, others might have regained FtsZ by lateral gene transfer (LGT) from other archaea, as shown by suspicious phylogenetic groups in FtsZ2, such as Micrarchaeota and Altiarchaeota branching within Euryarcheaota, or the monophyletic branching of Crenarchaeota and Halobacterota sequences (refer to question marks in Figure 4), both of which could represent phylogenetic artifacts (assuming that FtsZ has been inherited vertically between the species) or actual LGT events (as suggested by the latter case with full UFBoot2 support). Therefore, although vertical inheritance seems to be the main mode of evolution of FtsZ in archaea, LGT events between them should not be discarded. However, it is important to remark that we did not detect notorious LGT of *ftsZ* genes between archaea and bacteria.

The genomic contexts of the archaeal *ftsZ1* and *ftsZ2* genes are different from those of their bacterial counterparts, but they are also different from each other (Figure 3B). The gene *ftsZ1* has a conserved genome context, whereas the one of the *ftsZ2* is not as well conserved (Figure 3B). In contrast to what is observed in bacteria, the domains associated with genes found in the genome context of archaeal *ftsZ* are related to informational housekeeping genes, mainly related to protein biosynthesis, including ribosomal and t-RNA genes (especially for the genome context of *ftsZ1* but also in Crenarchaeota *ftsZ2*; Figure 3B). The genomic contexts of *ftsZ2* usually contain a gene bearing the RHH_1 Pfam domain, which probably functions as a transcriptional regulator of the CopG family (Acebo et al., 1998), suggesting that the transcriptional expression of the *ftsZ2* gene cluster could trigger a specific transcriptional regulation response.

### Homology at the C-termini suggests that CetZ and TubZ originated from archaeal FtsZ

**FIGURE 5:**
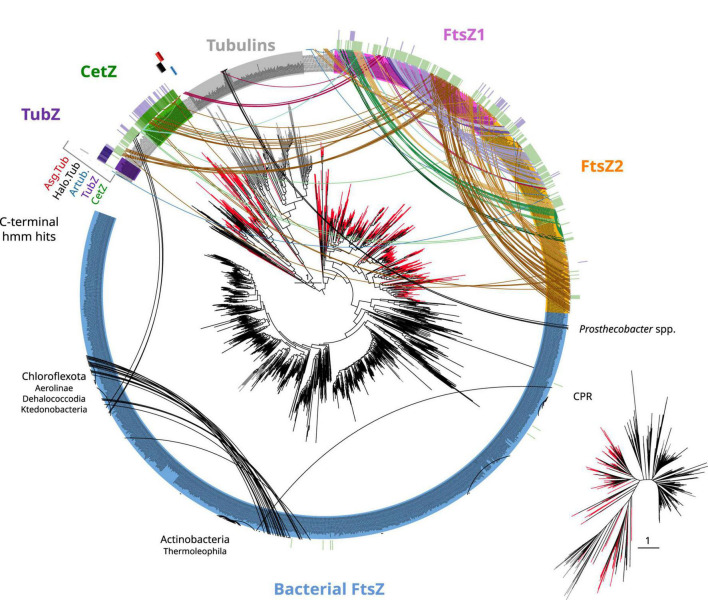
Tubulin/FtsZ-like paralogs in archaea and C-termini domain homologies. The phylogeny of Figure 4 was used to map the hits of HMMSEARCH of the C-termini of different phylogenetic groups (external colored heatmaps): CetZ, TubZ, artubulins, Halo.Tub, and Asg.Tub, showing the relationship between CetZ and archaeal FtsZ, as well as the overlap between CetZ and TubZ. Branches are colored by domain: black bacteria, red archaea, and gray eukaryota. Arcs connect sequences from the same organism showing the abundance and taxonomic diversity of FtsZ paralogs in archaea. Arc colors follow the legend of Figure 4. Inset represents an unrooted tree of the same phylogeny. Refer to the extended tree for annotations in Supplementary Figure 4.

**FIGURE 6:**
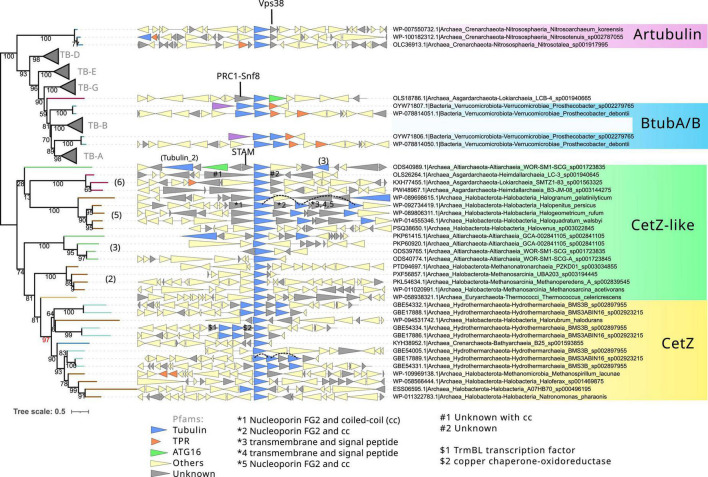
Phylogeny and genome context of the prokaryotic tubulin and CetZ-related sequences. The tree was rooted at the mid-point. The dataset for the phylogeny was selected manually, applying a gap trimming of 10%, resulting in a final MSA of 81 sequences and 485 positions. The tree was built using IQ-TREE, automatic model selection (LG + R5), and ultrafast bootstrap (UFBoot2). The number between parenthesis indicates the phylogenetic groups in Figure 4. Red bootstrap denotes the supported node defining the canonical CetZ proteins. Genes are colored according to the presence of Pfam domains, and genes without recognizable domains are colored in gray. Some of these latter were annotated with HHPred (indicated with #, *, $, and arrows). Discontinuous lines in the genome context panel show copies of the same CetZ(–like) that were omitted in this reconstruction.

Archaeal genomes often reflect functional expansions of the FtsZ/tubulin proteins (refer to arcs in Figure 5) as exemplified by the well-known CetZ and others, such as artubulins (Yutin and Koonin, 2012) and OdinTubulin from Odinarchaeota (Zaremba-Niedzwiedzka et al., 2017). In addition, we also identified other archaeal paralogs found in Asgardarchaeota (Asg.Tub) and another one in Halobacterota (Halo.Tub); although the phylogenetic positions of both were unstable, branching with tubulins (Figure 4) or with CetZ sequences (Figure 6; further discussed later).

CetZ was likely present in the Euryarchaeota ancestor as it is found in most classes, such as Archaeoglobi, Methanomicrobia, Halobacteria, Syntrophoarchaeia, and Hydrothermarchaeota (Figure 5). We found other CetZ-like proteins, including sequences from Halobacterota, Altiarchaeaota (DPANN), and sparse paraphyletic groups of Chloroflexota–Dehalococcoidia and Firmicutes–Bacilli/Clostridia (the latter was identified as TubZ; Figure 4).

As no recognizable domains were detected in the C-termini of the CetZ sequences (Figure 1), we attempted to clarify their evolutionary relationships by constructing protein models (hidden Markov model, HMM) of the C-termini of these proteins and performing hmmsearches against all our tubulin/FtsZ protein family collection (Supplementary Data). This CetZ_C HMM showed significant sequence similarities (e-value approximately 1e^–10^) with the other CetZ-like proteins (including bacterial Chloroflexota but not Firmicutes TubZ sequences) but also weaker similarities (e-value approximately 1e^–3^) with FtsZ, in particular with the archaeal sequences (see external green heatmap in Figure 5). CetZ_C HMM does not match with tubulins or archaeal tubulin-like proteins nor bacterial FtsZ. This possibility is more evident when visualizing the same tree unrooted (inset Figure 5). In this view, the closer proximity between the archaeal FtsZ sequences and tubulin-CetZ sequences is observed. Therefore, this phylogenetic signal, together with the homology at C-terminus, suggests that CetZ originated from the archaeal FtsZ.

The C-terminus of TubZ is substantially divergent, even between TubZ sequences. The HMM of CetZ_C did not match with that of Firmicutes TubZs, but the C-terminus HMM of TubZ matched with CetZ and archaeal FtsZ sequences (refer to external purple heatmap in Figure 5, e-value in the range of 1e^–2^), providing consistent support for the evolutionary relationship observed in the phylogenetic reconstructions (Figure 4). Thus, considering the potential antiquity of archaeal FtsZ compared with TubZ, it is likely that TubZ originated from CetZ or archaeal FtsZ sequences.

### Unclear origins of other archaeal tubulin-related proteins

Next, we inspected the C-termini of the archaeal groups branching basally to the tubulins, one of which is formed by Asgardarchaeota (Asg.Tub), another one by Halobacterota (Halo.Tub), and the third one by artubulins (Figure 4 and Supplementary Figure 4). The intermediary positions of these groups between CetZ and eukaryotic tubulins were stable when different phylogenetic methods were applied (Supplementary Figure 5). The monophyletic branching of Heimdallarchaeia and Lokiarchaeia is remarkable since it could illustrate the deep evolution of these paralogs in Asgardarchaeota. Here, it is important to mention that the scarcity of these groups translates into strict models that do not contain divergent sequences (note that the sequence conservation at the C-terminus is usually weak). The searches of their respective C-termini HMMs did not match with any other tubulin/FtsZ protein (refer to external red, black, and blue heatmaps in Figure 5 and Supplementary Data), even with each other. However, by inspecting the MSA of the selected sequences, we observed that their C-termini show limited similarity to CetZ sequences (Supplementary Figure 6). Conversely, we found that artubulins are more closely related to eukaryotic tubulins than to Halo.Tub and Asg.Tub since these contain important indels that characterize archaeal CetZ (and CetZ-like) sequences from eukaryotic tubulins (black highlight in Supplementary Figure 6), which is also supported by the mid-point rooting in the respective phylogeny (Figure 6). However, it is important to note that some of these CetZ-like sequences presented few conserved blocks at the N-terminus that resemble eukaryotic sequences (particularly in Altiarchaeota and different Halobacterota classes such as Methanosarcina, and Halobacteria, Halo.Tub, 5). Therefore, although our analyses suggest that Halo.Tub and Asg.Tub seems to be phylogenetically associated with the tubulins (Figure 4); inspections of the MSA and mid-point rooting of the selected set of sequences (Figure 6) argue against an apparent tubulin-like affiliation.

The genome context of these archaeal “tubulins like” is characterized by unknown genes that we manually annotated with HHPRED (Söding et al., 2005). We observed that most of these contain coiled-coil regions with different functions (refer to gray triangles in Figure 6). In cases such as Halo.Tub, their genome context contains several copies in a quasi-tandem disposition. In contrast, artubulin from *Nitrososphaera korensis* is associated with a potential Snf7 protein upstream, as previously observed (Yutin and Koonin, 2012). This abundance of unknown and unrelated genes could illustrate the diversity of molecular systems and functions in which these archaeal CetZ/Tubulin homologs are involved.

Therefore, the divergence of their C-termini, together with the sparse distribution of each phylogenetic group, shows the phylogenetic positions of these Halo.Tub and Asg.Tub in relation to each other and with eukaryotic tubulins must be interpreted cautiously. These proteins appear to be more closely related to CetZ sequences than eukaryotic tubulins. The genomic context between the CetZs and these CetZ-like sequences is not related (Figure 6), hindering inferences regarding the origin of these proteins. Indeed, the idea of LGTs between archaea, followed by neofunctionalization, should not be discarded for these specific proteins. Nevertheless, our results suggest that artubulins are the only ones of these proteins that could be related to ancestral eukaryotic tubulins, as previously pointed out (Yutin and Koonin, 2012).

### Prokayotic tubulins are closely related to α/β-tubulins

The monophyletic branching of eukaryotic tubulins (Figure 6) confirms that eukaryotic tubulins had a single origin and suggests that its paralogs (α, β, γ, δ, ε, and ζ) were already established in the LECA as previously proposed (Findeisen et al., 2014). Other divergent tubulin-related proteins such as Misato, were not included in this analysis because of their strong sequence divergence. Tubulins have a very restricted distribution in prokaryotes and are only found in one bacterial species and one archaeal metagenome. OdinTubulin is only found in Odinarchaeota from the Asgardarchaeota phylum and a pair of tubulins, BtubA and BtubB, is present in various *Prostecobacter* species from the Verrucomicrobiota phylum. These prokaryotic sequences branch basally and paraphyletically to the α- and β-tubulins, although BtubA and BtubB can also branch basally to α- and β-tubulins, respectively (Figures 4, 6). These phylogenetic instabilities of BtubA/B have been observed previously (Findeisen et al., 2014). In our reconstructions, their monophyletic branching was exclusively observed in the inclusive trimming, removing only gap positions (Supplementary Figure 5 right panel). In contrast, using a stricter trimming with BMGE, BtubA and BtubB were paraphyletic (Supplementary Figure 5 right panel; note that the topology of eukaryotic tubulins also differed and branched monophyletically to the β/γ group in BMGE trimming). In contrast, using an inclusive trimming (trimAl -gt 0.10) and considering only tubulin and CetZ(–like) sequences, the paraphyly of BtubA/B was again recovered (Figure 6). Therefore, the mono/paraphyly of BtubA/B seems to be sensitive to the trimming methods and the diversity of sequences included in the MSA. Similarly, the locations of the OdinTubulin in our reconstructions (Figures 4, 6) differed from those obtained in previous studies, in which the Odinarchaeota sequence branched basally to the whole eukaryotic tubulin family (Zaremba-Niedzwiedzka et al., 2017), instead of basally to α- and β-tubulins. These differences could be due to different parameters of the reconstructions (taxonomic sampling, the use of different outgroups, or different methods for the MSA trimming), showing the phylogenetic instability of these prokaryotic tubulins and, therefore, the difficulty in inferring their evolutionary placement. Nevertheless, from our reconstructions, it is clear that OdinTubulins and BtubA/B are closer to α- and β-tubulins, in agreement with recent analyses (Akıl et al., 2022).

In eukaryotes, the α- and β-tubulins generally form 13 subunits filaments as imposed by the nucleation of the γ-tubulin ring composed of 13 γ-tubulins (Chaaban and Brouhard, 2017; Cavalier-Smith and Chao, 2020). The *Prosthecobacter*’s BtubA/B microtubules are formed by four or five protofilaments, presenting heterodimer polarity which can polymerize without the need of the microtubule processing centers, tubulin γ, never found in prokaryotes. In addition, *btubA/B* genes form an operon together with the *btubC* gene, a “bacterial kinesin light chain” that has been shown to stabilize the filament (Deng et al., 2017). Therefore, the functional similarities between BtubA/B and α- and β-tubulin supports their close relationships.

In contrast to the dual BtubA/B, there is a unique OdinTubulin in the Odinarchaeota metagenome sequenced from hydrothermal environments (Zaremba-Niedzwiedzka et al., 2017). Despite the possible incompleteness of the Asgardarchaeota metagenomes, the sequencing of several Asgardarchaeota clades (Liu et al., 2021) confirmed that this OdinTubulin is still uniquely found in this archaeon. *In vitro* and at high temperatures, this OdinTubulin forms tubules with short curved protofilaments coiling around the tubule circumference, more similar to FtsZ, rather than running parallel to its length as in eukaryotic microtubules (Akıl et al., 2022). Nevertheless, the genome context of this OdinTubulin displays interesting features related to eukaryotes. A hypothetical protein showing similarity to Prc1 at the N-terminus and Snf8 at the C-terminus (containing coiled-coil segments) is located upstream of the OdinTubulin gene. Prc1 is a key regulator of cytokinesis that cross-links antiparallel microtubules, while Snf8 is a member of the ESCRT-II system. Downstream of the OdinTubulin, we found a gene containing coiled-coil segments weakly related to the ATG16 Pfam domain, which belongs to the eukaryotic autophagy system. However, according to the NCBI annotation, this gene is annotated as chromosome partitioning protein Smc. The two genes surrounding the OdinTubulin are specific to this genome as protein searches do not retrieve any homolog sequences in the three domains. Together, these observations suggest that this OdinTubulin is specific to species related to Odinarchaeota and that it could be functionally related to a eukaryotic-like system involved in endomembrane system and cell division (exemplified by the hypothetical prc1-snf8 gene), in agreement with its phylogenetic position.

Therefore, even though BtubA/B and OdinTubulin are closely related at the sequence level, as they are associated with α- and β-tubulins, they seem to be involved in different molecular systems or complexes, where the BtubA/B pair functions as eukaryotic-like microtubule filaments and OdinTubulin could function as monomeric filaments in combination with the Pcr1-Snf8 protein.

## Discussion

In this study, we investigated the early origin and diversification of the main members of the FtsZ/tubulin protein family, which was already present in the LUCA. We inferred that FtsZ was present in the LBCA and LACA, although, in the latter, FtsZ was duplicated before the diversification of the Archaea domain. The differences in the gene organization around the *ftsZ* gene in archaeal and bacterial genomes reveal different evolutionary histories. Bacterial *ftsZ* genes are associated with cell division and peptidoglycan synthesis genes, while one of the archaeal *ftsZ*s is mainly associated with genes involved in protein biosynthesis (t-RNA, ribosomal proteins, etc.). The latter suggests that the expression of the archaea FtsZ gene (mainly FtsZ1) is concurrent with an increase in protein biosynthesis, perhaps necessary for preparing the cell division. In contrast, peptidoglycan biosynthesis genes are nearly absent in archaea, except for a few Euryarchaeota, such as Methylobacteriales and Methylopyrales, that are able to produce peptidoglycan-like cell walls made up of pseudomurein, and whose biosynthesis has a common origin with the bacterial murein, but not necessarily reflecting an LGT from bacteria (Subedi et al., 2021). Nevertheless, the gene cluster involved in the biosynthesis of pseudomurein does not contain *ftsZ* (manually checked), suggesting that the duplication of FtsZ and acquisition of pseudomurein in these Euryarchaeaota orders were independent. In fact, although intradomain LGT of FtsZ can not be discarded, it is important to note that we did not detect any case of FtsZ LGT between bacteria and archaea. Therefore, this scenario of different genome landscapes around *ftsZ* genes is possibly a reflection of the evolutionary pressures that happened during the divergence between Bacteria and Archaea, such as the loss or acquisition of peptidoglycan biosynthesis.

Our analyses also demonstrate that the C-termini of these proteins contain enough evolutionary signals to solve their relationships. In particular, we show that the archaeal CetZ (mainly found in the Euryarchaeota supergroup) and the plasmidic TubZ (found in Firmicutes), most likely originated from archaeal FtsZ. This last observation regarding TubZ could clarify previous claims regarding the affiliation of TubZ to prokaryotic FtsZ (Findeisen et al., 2014). In contrast, the exclusiveness of the different C-termini domains in CetZ-like proteins, together with their unrelated genome context, suggests a complex evolutionary history of these proteins. In the MSAs, we observed consistent evidence to at least differentiate Halo.Tubs and Asg.Tubs from artubulins, and to define artubulins as members of the eukaryotic tubulin protein family—arguing thus against previous assumptions stating a close relationship between TubZ and artubulins (Findeisen et al., 2014). Given that the evolution of these sequences is irregular in archaea (and in bacteria), LGT events between them could be the most plausible explanation for such a distribution. However, given the close proximity of archaeal paralogs to eukaryotic tubulins, which probably originated from archaeal FtsZ, our data still suggest an archaeal origin of tubulins, as previously suggested (Yutin and Koonin, 2012).

Prokaryotic tubulins, such as BtubA/B and OdinTubulin, seem to be closely related to α- and β-tubulins. This could be an indication of the origin and diversification of eukaryotic tubulins from α- and β-tubulin paralogs, and consequently, the basal position of the rest of the tubulins could be the result of strong divergence due to subfunctionalization. In fact, the monophyletic branching of BtubA/B in some of our reconstructions could reflect a reminiscence of the early diversification of tubulins by gene duplications, although further analyses are required to test this assumption. Nevertheless, while prokaryotic tubulins are probably involved in different cellular processes, there is no doubt that they are similar to the ancestral eukaryotic molecular systems or complexes. Altogether, our analyses revisit the evolution of the main members of the FtsZ/tubulin protein family, providing evidence of its early origin and diversification across the three domains of life.

## Materials and methods

### Collection of FtsZ/tubulin homologs

To collect homologous sequences from the tubulin/FtsZ protein family, we performed HMMSEARCH (HMMER 3.1b2) (e-value threshold 1e^–3^; Prakash et al., 2017) against a local database comprising all the NCBI genomes taxonomically annotated with GTDB (release bac120 and arc122; Parks et al., 2018), plus 36 diverse eukaryotes (24,664 taxa in total). As a query, we used the Tubulin Pfam model (PF00091) spanning the N-terminus as it is the commonly shared domain of this family, for both FtsZ, tubulins, and derivatives. This search also detected FtsZ-like sequences (Makarova and Koonin, 2010), containing the Tubulin_2 Pfam model (PF13809) instead. The scattered and sparse taxonomic distribution of this FtsZ-like (Tubulin_2), together with their irregular domain architecture, that is, they do not have the FtsZ_C nor Tubulin_C Pfam domains but have important N/C-terminus extensions (in Planctomycetes a protein kinase domain is found at the N-terminus). Their sequence divergence (to tubulin/FtsZ family members but also between them) suggested that these proteins had a different evolutionary history to the canonical tubulin/FtsZ protein family (Makarova and Koonin, 2010). Thus, to obtain more accurate multiple sequence alignments (MSAs) and phylogenies, we exclude these sequences from our main reconstructions.

### Dataset construction, MSA, and phylogenies

To define the main tubulin/FtsZ subfamilies, we aligned all the sequences (45,929 sequences) with MAFFT v7.310 (Katoh and Standley, 2013), applied gap trimming using trimAl 1.4.22 (–gt 0.7; Capella-Gutiérrez et al., 2009), removed sequences with lower alignment coverage than 60%, built a Fasttree 2.1.10 (Price et al., 2010), and selected the branches of interest to build different datasets (FtsZ, tubulin-like proteins or to exclude FtsZ-like sequences). To avoid the over-representation of some taxa and build taxonomically balanced datasets, we removed redundant sequences (using CD-HIT; Fu et al., 2012) by taxonomic classes (following the GTDB taxonomy) and applied different cut-offs depending on the number of sequences (from 20 sequences, 95% identity, up to >250, 55%).

From these reduced datasets, we built different datasets for specific purposes: that is, focused on prokaryotic FtsZ (Figure 2), all tubulin/FtsZ and homologous proteins with a special focus on archaea (Figures 4, 5), and tubulin/CetZ-like proteins (Figure 6). All trees were visualized and annotated using iTOL (Letunic and Bork, 2019). Raw data for the MSAs and respective phylogenetic trees are provided in Supplementary Data.

For the phylogenies of bacterial FtsZs (Figure 2), we randomly selected one sequence from each taxonomic order from the FtsZ prokaryotic dataset (note that some taxa such as Verrucomicrobiota, CPR were manually increased by selecting representative sequences). Then, we removed spurious sequences based on the visualization of the MSA (trimAl -gt 20) and the respective phylogenetic tree (Fasttree). The final set of sequences was aligned with MAFFT-linsi, and three filtering methods were applied: gap-trimmed alignment (–gt 0.2), BMGE-1.12 (–h 0.55 -m BLOSUM30), using trimAl (–gt 0.7). Due to some miss-aligned regions at the C-terminus in FtsZ from Verrucomicrobia, the manually trimmed alignment was re-aligned as follows: We ran MAFFT-Linsi excluding CPR-Verrucomicrobia divergent sequences, and these later ones were aligned independently; then, both MSAs were treated with trimAl -gt 0.2 and merged with MAFFT (–merge option). Each trimming method had two sub-datasets: one including bacterial and archaeal FtsZs (BA), and the other one containing exclusively bacterial FtsZs (B). From the manually trimmed alignments, B MSA was obtained by removing archaeal sequences from BA MSA, while in BMGE- and trimAl-gt70-trimming MSAs, B, and BA MSAs were aligned independently through MAFFT-Linsi.

For the phylogeny including FtsZ, tubulins, and others (Figures 4, 5), the dataset was constructed by a selection of representative bacterial FtsZs and eukaryotic tubulins, plus an archaeal selection based on redundancy filtering and recovering the discarded paralogs from the remaining taxa. Then, this set of the sequence was aligned with MAFFT-Linsi and trimmed with trimAl (–gt 0.2) and BMGE (the same options as mentioned earlier).

For the phylogeny of tubulins and CetZ(–like) sequences (Figure 6), we manually selected a set of representative sequences branching basally to eukaryotic tubulins extracted from the tree of Figure 3.

Phylogenies were inferred using maximum-likelihood methods with IQ-TREE (Nguyen et al., 2015). We obtained branch support with ultrafast bootstrap (UFBoot2, option -B 1000; Hoang et al., 2018), and the evolutionary models of each set of the sequences were automatically selected using ModelFinder (Kalyaanamoorthy et al., 2017), following the BIC criterion (–m MFP). In addition, we also run UFBoot2 + NNI bootstraps (-B 1000 -bnni options), testing complex evolutionary models such as LG4X (–m LG4X) or C10-30 (–mset LG -madd LG + C10, LG + C20, LG + C30, LG + C10 + R + F, LG + C20 + R + F, LG + C30 + R + F –score-diff all).

### Genomic context

To analyze the genome contexts of the tubulin/FtsZ protein family, the DNA sequence corresponding to 10 Kb surrounding (5 kb up/downstream) each of the respective coding genes was extracted. In that fragment, genes were predicted using Prodigal (Hyatt et al., 2010), with default parameters, and the predicted proteins annotated with HMMSCAN (Potter et al., 2018) on the Pfam database (Finn et al., 2011). The abundance of Pfam domains in these proteins was then quantified, counting only the presence and not the repetitions inside the same protein or genome context, that is, multiple copies of the same domain in one gene or multiple gene copies in one genome context was only counted once.

### Definition of C-termini protein models and searches

To build the protein models (hidden Markov model, HMM) of the C-termini of the different proteins of interest (CetZ, TubZ, artubulins, Halo.Tub, and Asg.Tub), the sequences were aligned individually, and soft gap trimming was applied with trimAl (–gt 0.1). Then, the C-termini fragments were extracted starting from the end of the Tubulin Pfam domain (N-terminus domain). Protein models were made using HMMBUILD (Potter et al., 2018) and were then used to perform hmmsearches against our dataset of the FtsZ/tubulin protein family without an e-value threshold. Raw data for the construction of protein models and respective searches are provided in Supplementary Data.

## Data availability statement

The original contributions presented in this study are included in the article/Supplementary material, further inquiries can be directed to the corresponding authors.

## Author contributions

DD: funding acquisition and supervision. CS-M and DS-N: investigation and formal analysis. All authors contributed to the article and approved the submitted version.
