# Supplementary material for: Early origin and evolution of the FtsZ/tubulin protein family
Source: Front Microbiol. 2023 Jan 10;13:1100249. doi: 10.3389/fmicb.2022.1100249 (PMC9871819; doi:10.3389/fmicb.2022.1100249)
Supplement: Supplementary file 1 [file Data_Sheet_1.PDF]

SUPPLEMENTARY INFORMATION

Early origin and evolution of the FtsZ/Tubulin protein family

Carlos Santana-Molina[1,2*], DMaría del Saz-Navarro[1] Damien P. Devos[1*]

[1] Centro Andaluz de Biología del Desarrollo (CABD)-CSIC, Universidad Pablo de Olavide. Spain.
[2] Department of Marine Microbiology and Biogeochemistry, NIOZ, Royal Netherlands Institute for Sea Rechear, and Utrecht University, Netherlands.

* Correspondence author e-mail:
D.P.D. (damienpdevos@gmail.com), C.S-.M. (csantmol@gmail.com)

Supplementary figures legends

Supplementary Figure 1. Phylogenetic analyses on prokaryotic FtsZ testing different trimming methods and evolutionary models. Branches are colored according to the phyla and black dots indicate bootstraps higher than 90% obtained with -bnni option in IQ-TREE. Yellow and purple highlights indicate Terrabacteria Gracilicutes phyla, respectively, and no highlight indicates mix of supergroups. The parameters for each tree reconstruction, and the multiple sequence alignment (MSA) features are indicated. The reconstructions above are based on a manual trimming MSA, while the reconstructions below are based on automatic trimming. Raw data for these analyses are provided in Supp. DATA.

→ This figure shows the phylogenetic instability of bacterial phyla in FtsZ phylogenies obtained from different trimming and phylogenetic methods. However, most of reconstructions recover Terrabacteria and Gracilicutes split suggesting that that vertical evolution is the main mode of evolution of FtsZ in Bacteria. Verrucomicrobia sequences are the most divergent bacterial FtsZ. The second copy of CPR, is found within CPR's FtsZ in Linsi-BMGE-LG+C20+R+F. The paraphyletic/monophyletic branching of the divergent paralogs of CPR and verrucomicrobial FtsZ shows their tendency to provoke long branch attraction artefacts. Likewise, it is important to remark that when archaeal FtsZ sequences are included, different attraction are observed, suggesting that archaeal FtsZs promote long branch attraction artefacts in these reconstructions.

Supplementary Figure 2. Effect of phylogenetic support in FtsZ phylogeny including (BA_) and excluding the (B_) archaeal FtsZs. Phylogenies in row show the topology removing those nodes with lower bootstrap than 95, 85 and 60%. Branches are colored according to the phyla. Bootstrap support represents UFBoot2 values.

→ This figure shows the topologies removing nodes at different threshold of UFBOOT2 values: 95, 85 and 60%. At 95% it is possible to observe the split of Gracilicutes and Terrabacteria, providing further support for the ancestral evolution of FtsZ in Bacteria. However, the relationships between bacterial phyla become more meaningful when relax threshold at 85%. At 60%, the relationship between bacterial phyla can be further observed, but we consider these as low UFBoot2 values. It can also be observed that archaeal FtsZs attract bacterial sequences hindering the monophyly of bacterial groups like Verrucomicrobia and Bacteroidetes FtsZ among others. The difference between these trees and trees at the top in Supplementary Figure 1, is that these ones are preformed using empirical models and UFBoot2 estimation, while in previous figure, trees are inferred with complex evolutionary models and using -bnni option for UFBoot2 optimization.

Supplementary Figure 3. Extended view of the bacteria FtsZ tree shown in Figure 1 and Supplementary Figure 2. Bootstrap support represents UFBoot2 values.

Supplementary Figure 4. Extended view of the tubulin/FtsZ protein family of phylogeny shown in Figure 3 and 4. Branches are coloured according to the archaeal phyla, arcs connect paralogs in the same organisms, and heatmaps, shows the HMMSEARCH hits of the respective C-termini. Bootstrap support represents UFBoot2 values.

→ This figure shows the duplication of thetubulin/FtsZ proteins, in between FtsZ copies, but also the relationship between these FtsZs and the related proteins, such as CetZ or TubZ.

Supplementary Figure 5. Phylogenetic analyses on tubulin/FtsZ protein family centered on archaea and prokaryotic tubulins. Branches are colored according to the archaeal phyla, eukaryotic sequences in gray and bacterial sequences in black. Black dots indicate bootstraps higher than 90 obtained with -bnni option in IQ-TREE. A) Phylogeny using a MSA removing only gap positions and B) phylogeny removing heterogeneous positions using BMGE. Left and central panels show trees built with empirical models and central panel trees conducted with complex evolutionary models. Right panels show zooms in into the different topologies of prokaryotic tubulins in both reconstructions.

→ This figure shows the stable phylogenetic position of Artubulins, Halo.Tub and Asg.Tub branching between CetZ and eukaryotic tubulins. In addition, this figure also shows the monophyletic and paraphyletic branching of BtubA/B when using an inclusive trimming (A), and strict trimming (B). The long branch attraction of verrucomicrobial FtsZ can also be observed.

Supplementary Figure 6. Phylogeny with mid-point rooting (left) and respective MSA (right), of selected eukaryotic and prokaryotic tubulins, and CetZ (-like) sequences. Indels highlighted with black boxse at the top shows similarities between tubulins and CetZ (-like) sequences. Long thin black line, separate CetZ sequences from other sequences discussed in the text: Halo.Tub and Asg.Tub.

→ This figure shows the similarities of Halo.Tub and Asg.Tub sequences with CetZ sequences despite their intermediary positions between CetZ and eukaryotic tubulins. This relationship is further supported by the mid-point rooting of the respective phylogeny.

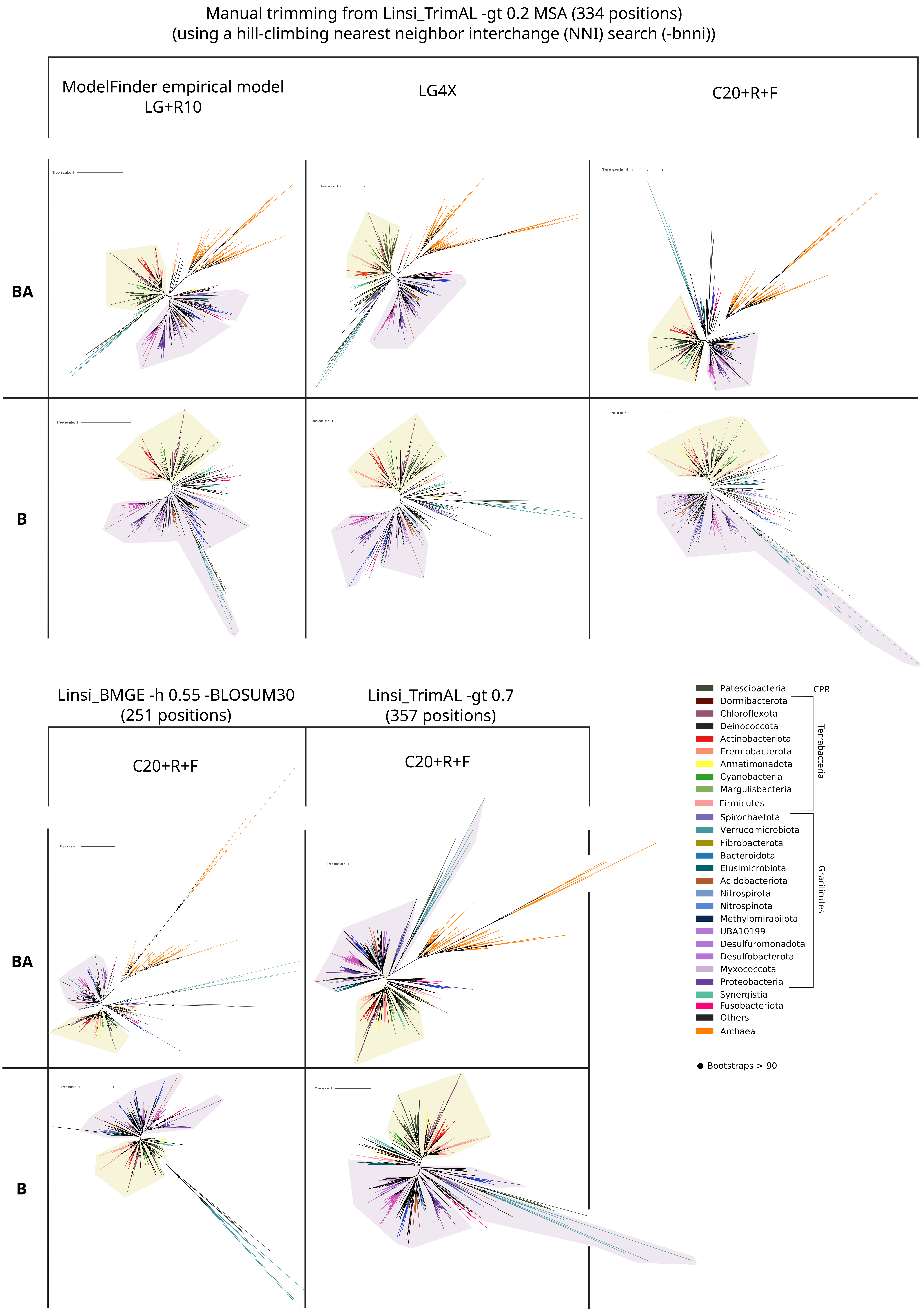

Supplmentary Figure 1.

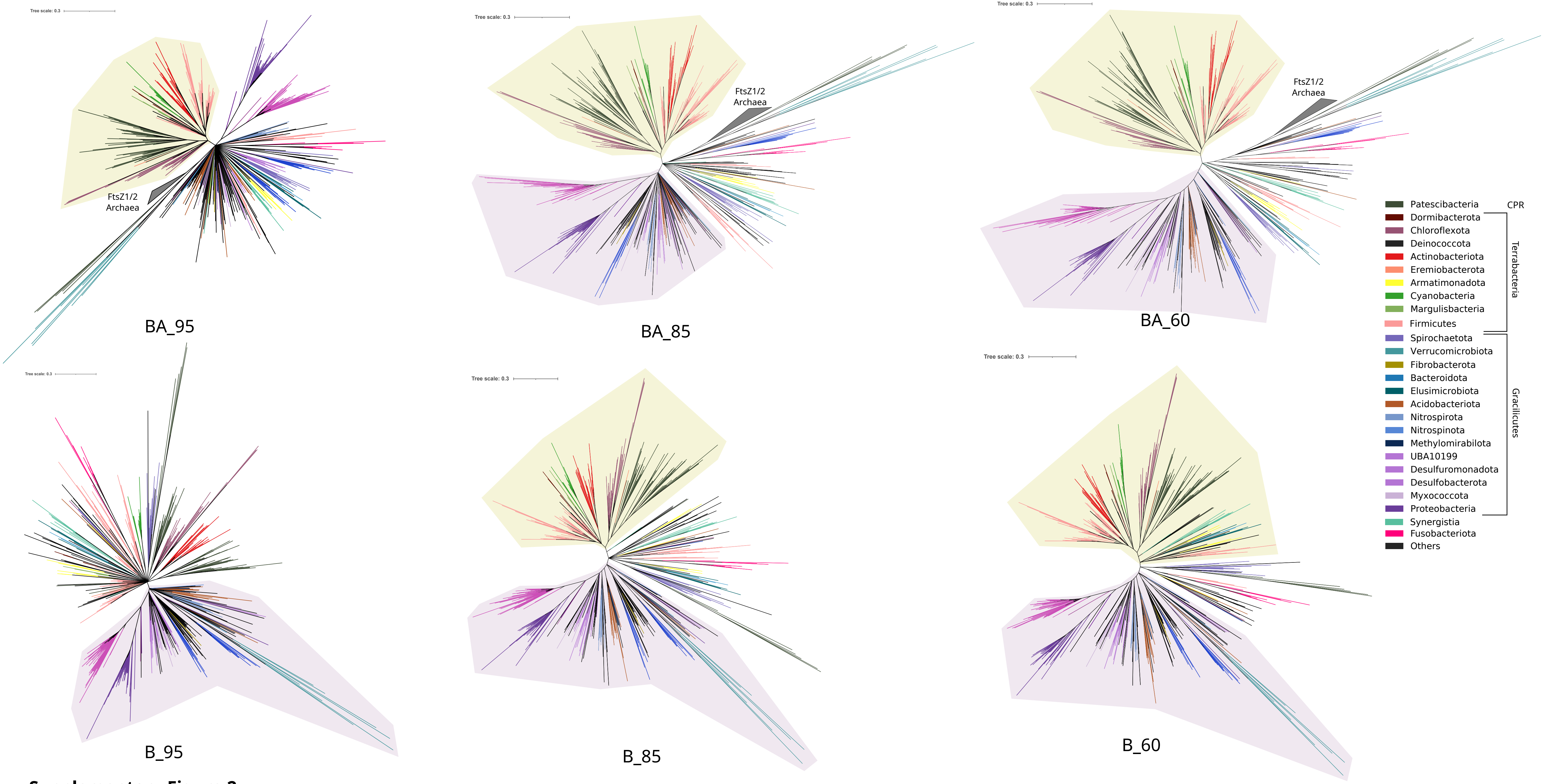

Supplementary Figure 2.

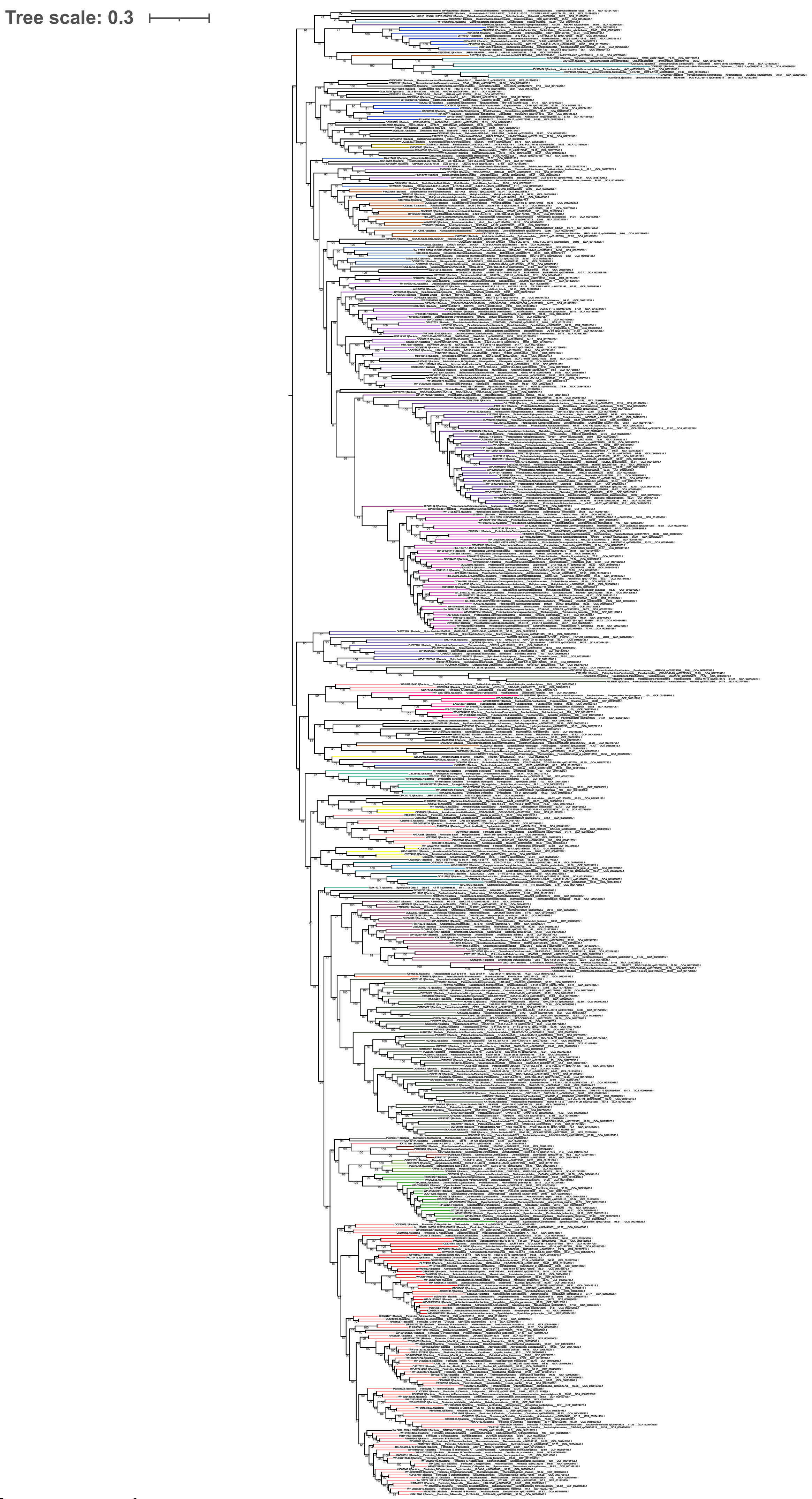

Supplementary Figure 3.

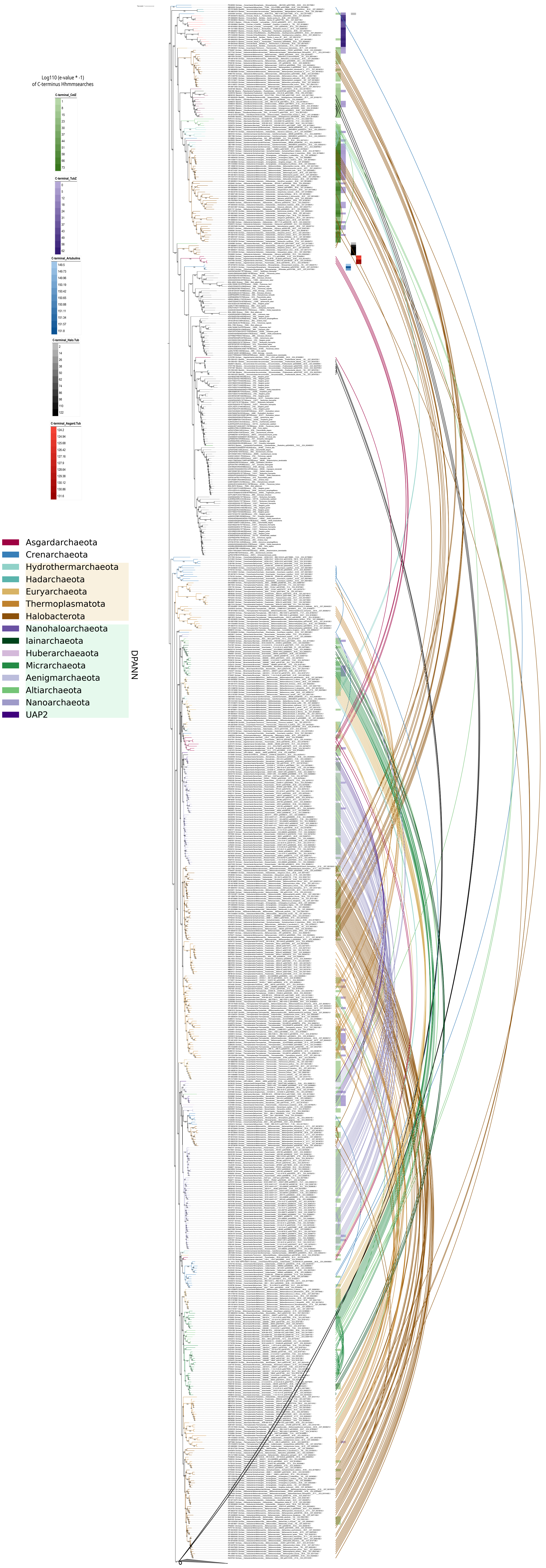

Supplementary Figure 4.

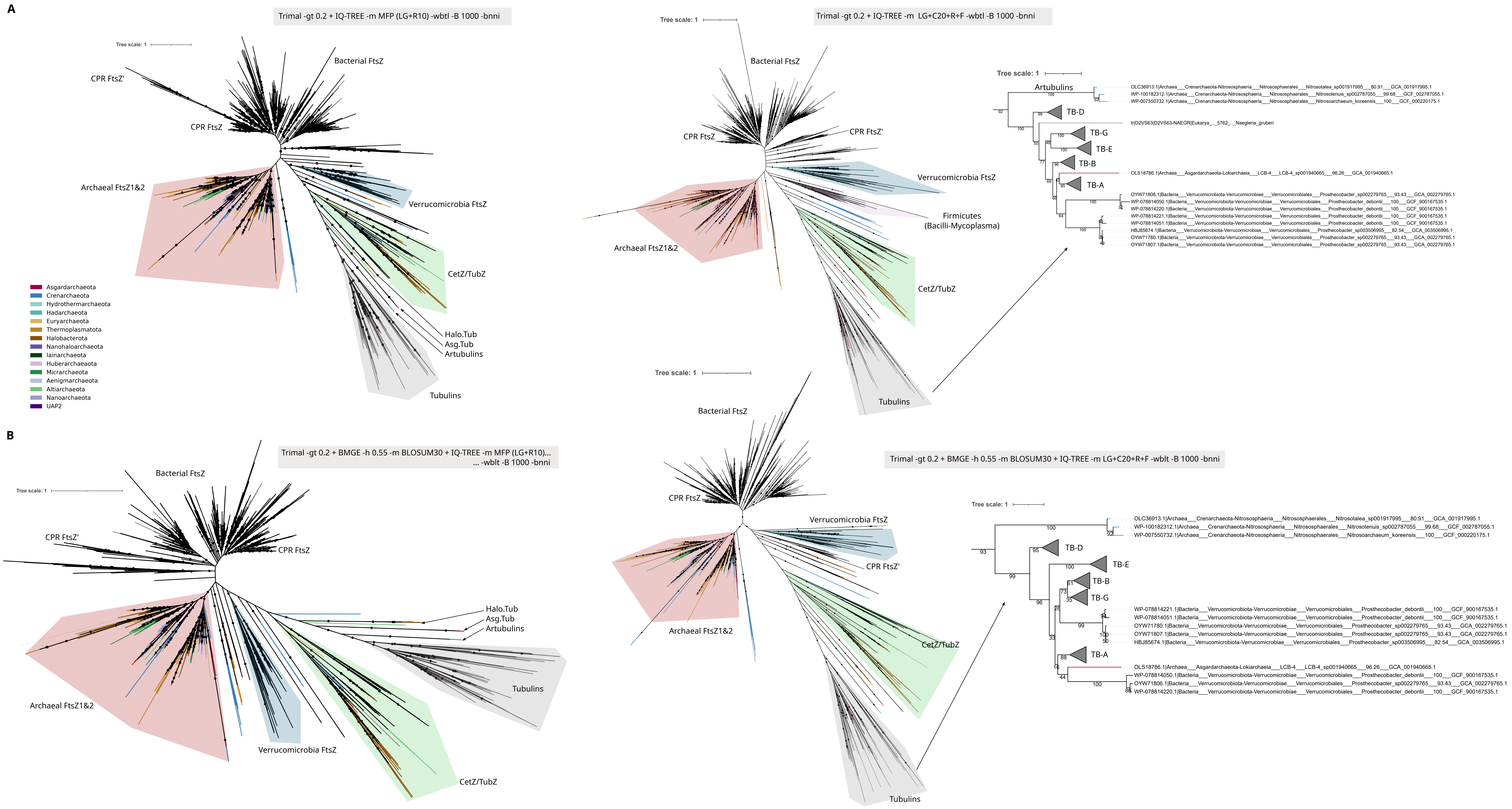

Supplementary Figure 5.

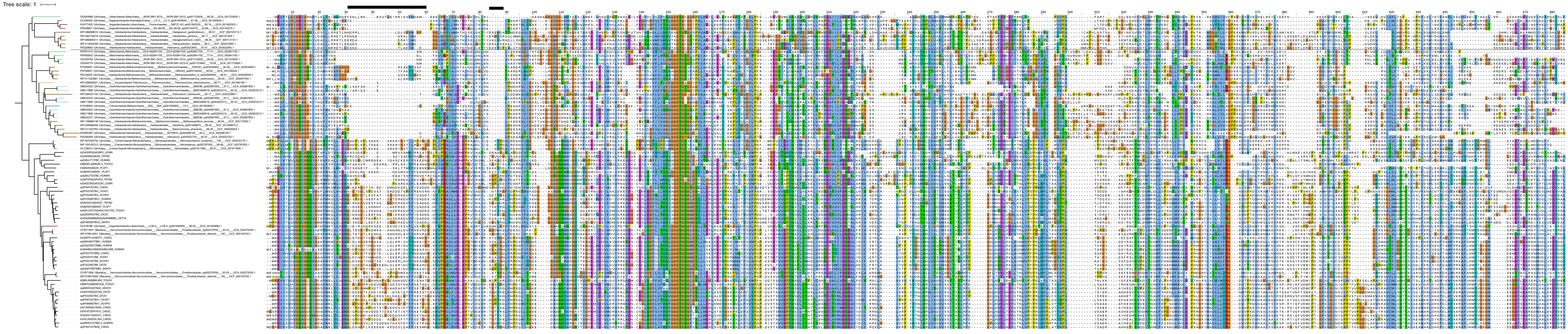

Supplementary Figure 6.
